# Molecular Mechanisms Underlying IL-33-Mediated Inflammation in Inflammatory Bowel Disease

**DOI:** 10.3390/ijms24010623

**Published:** 2022-12-30

**Authors:** Ioanna Aggeletopoulou, Efthymios P. Tsounis, Christos Triantos

**Affiliations:** Division of Gastroenterology, Department of Internal Medicine, University Hospital of Patras, GR-26504 Patras, Greece

**Keywords:** intereukin-33, ST2 receptor, Crohn’s disease, ulcerative colitis, inflammatory bowel disease, pathogenesis

## Abstract

Interleukin-33 (IL-33) is a cytokine defined by its pleiotropic function, acting either as a typical extracellular cytokine or as a nuclear transcription factor. IL-33 and its receptor, suppression of tumorigenicity 2 (ST2), interact with both innate and adaptive immunity and are considered critical regulators of inflammatory disorders. The IL-33/ST2 axis is involved in the maintenance of intestinal homeostasis; on the basis of their role as pro- or anti-inflammatory mediators of first-line innate immunity, their expression is of great importance in regard to mucosal defenses. Mucosal immunity commonly presents an imbalance in inflammatory bowel disease (IBD). This review summarizes the main cellular and molecular aspects of IL-33 and ST2, mainly focusing on the current evidence of the pro- and anti-inflammatory effects of the IL-33/ST2 axis in the course of ulcerative colitis and Crohn’s disease, as well as the molecular mechanisms underlying the association of IL-33/ST2 signaling in IBD pathogenesis. Although IL-33 modulates and impacts the development, course, and recurrence of the inflammatory response, the exact role of this molecule is elusive, and it seems to be associated with the subtype of the disease or the disease stage. Unraveling of IL-33/ST2-mediated mechanisms involved in IBD pathology shows great potential for clinical application as therapeutic targets in IBD treatment.

## 1. Introduction

Inflammatory bowel disease (IBD) is a term mainly used to describe two chronic autoimmune gastrointestinal diseases: ulcerative colitis (UC) and Crohn’s disease (CD). These disorders are characterized by uncontrolled adaptive and innate immune responses resulting in sustained mucosal inflammation. The mechanisms underlying the IBD pathogenesis is a topic of great interest [1,2]. The interplay between environmental factors and genetic predisposition, defects in the gut barrier integrity, changes in the gut microbiota community, and impaired regulation of immune responses are the dominant contributing factors related to pathogenesis and development of IBD [3,4,5,6,7,8,9,10]. The complex interaction between the adaptive and innate immune responses is mediated by various cytokines; disturbance in this crosstalk may result in the instigation and propagation of mucosal inflammation. Interleukin (IL)-1, an integral mediator of innate immune responses, constitutes a group of 11 proinflammatory and anti-inflammatory cytokines (seven ligands with agonist function (IL-1α, IL-1β, IL-18, IL-33, IL-36α, IL-36β, and IL-36γ) and four antagonist ligands (IL-1 receptor antagonist (IL-1Ra), IL-36Ra, IL-37, and IL-38) [11]. The precursor protein length and the N-terminal segments for each precursor, lead to the segregation of the IL-1 group into three subfamilies: IL-1, IL-18, and IL-36. The IL-1 subfamily is composed of the cytokines IL-1α, IL-1β, and IL-33, and it carries the largest segments [11]. This family critically contributes to the regulation of inflammatory responses, repair of the impaired tissue, and maintenance of intestinal homeostasis by the stimulation of signaling pathways involved in innate immune responses [12]. Specifically, IL-33 acts as a major mediator of tissue damage and an interface between innate and adaptive immunity. Dysregulation of IL-33 and its receptor signaling have been strongly implicated in a variety of inflammatory diseases, including IBD [13,14,15,16], highlighting this cytokine as a critical molecule of mucosal immunity.

This review provides an overview of the cellular and molecular characteristics of IL-33 and its receptor, suppression of tumorigenicity 2 (ST2), describes the current evidence on the pro- and anti-inflammatory effects of IL-33/ST2 axis in the course of UC and CD, and discusses the molecular mechanisms underlying the complex association between the IL-33/ST2 signaling axis and IBD pathogenesis.

## 2. IL-33 Biological Function

**Figure 1:**
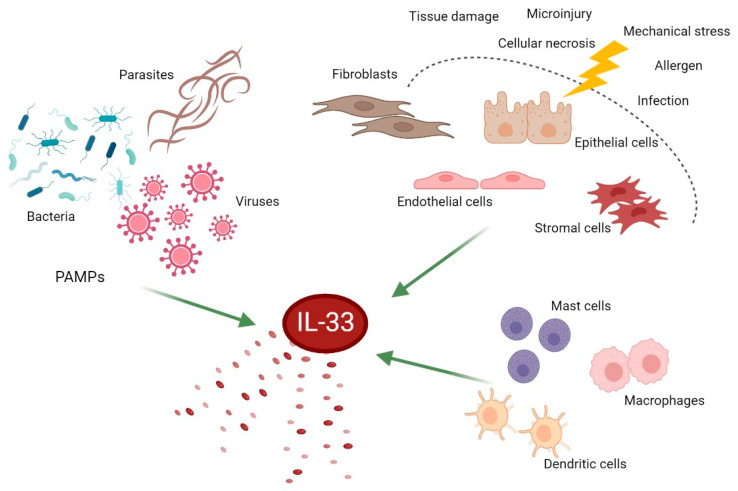
Schematic representation of IL-33-producing cells. This figure was created with BioRender (https://biorender.com (accessed on 14 December 2022)). IL-33, interleukin-33; PAMPs, pathogen-associated molecular patterns.

IL-33, also known as IL-F11, was originally discovered as an activator of T helper type 2 (Th2) cells [17,18]. IL-33, a 30 kDa protein, composed of 270 amino acids, plays an important role in the maintenance of tissue homeostasis and repair, in type 2 immune responses, in inflammation induced by allergic and nonallergic triggers, and in viral infections and malignancies [19,20]. This cytokine is commonly released in response to apoptosis, cellular damage, mechanical stress, or immune response stimulation as a full-length biologically active molecule, and it is considered a member of the “alarmins” family [20] (Figure 1).

**Figure 2:**
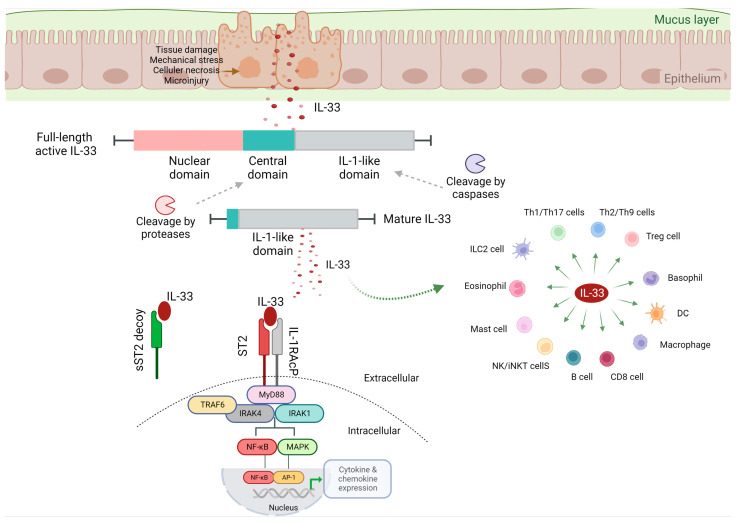
Activation of IL-33/ST2 signaling. Full-length IL-33 is composed of an N-terminal nuclear domain and a C-terminal IL-1-like cytokine domain, divided by a central domain. IL-33 signals through a great variety of immune cells promoting their function. Binding of IL-33 to sST2 decoy receptor prevents the ST2/IL-33 signaling, whereas binding of IL-33 to ST2 results in the activation of the transcription factor NF-κB and the MAP kinases, leading to related-gene transcription. This figure was created with BioRender (https://biorender.com (accessed on 29 November 2022)). IL-33, interleukin-33; ST2, suppression of tumorigenicity 2; sST2, soluble ST2; IL-1RAcP, IL-1 receptor accessory protein; MyD88, myeloid differentiation primary response 88; TRAF6, tumor necrosis factor receptor-associated factor 6; IRAK 1, interleukin receptor-associated kinase; NF-κB, nuclear factor κB; MAPK, mitogen-activated protein kinases; AP-1, activator protein 1; Th1 cells, T helper 1 cells; Treg cells, T regulatory cells; DC cells, dendritic cells; CD8 cells, cytotoxic T cells; NK cells, natural killer cells; iNKT cells, invariant natural killer T cells; ILC2 cells, group 2 innate lymphoid cells.

Growing evidence has highlighted IL-33 as a pleiotropic cytokine, which not only activates the Th2 cells but also induces Th1 cells, group 2 innate lymphoid cells (ILC2s), and T regulatory cells (Tregs) [17,18]. Beyond adaptive immunity, IL-33 is expressed by a wide variety of cell types including tissue-derived cells, vascular endothelium, epithelial barrier, stromal fibroblasts, and antigen-presenting cells (APCs), upon encountering microbial infection, exposure to allergens, or tissue damage [17,18]. IL-33 release leads to the stimulation of myeloid differentiation primary response 88 (MyD88)-dependent signaling pathways in cells expressing the interleukin 1 receptor-like 1, also known as IL1RL1 and ST2 [21] (Figure 2).

### 2.1. IL-33/ST2 Signaling

ST2 is a full-length, membrane-spanning receptor that exists in two different forms as splice variants, the soluble form (sST2) and the membrane bound form. The sST2 form acts as a decoy receptor and is responsible for the sequestering of free IL-33, whereas ST2 induces the MyD88/nuclear factor κB (NF-κB) signaling pathway to promote the function of immune cells [22]. ST2 is constitutively expressed in various immune cells, including Th1 cells, Th2 cells, cytotoxic T cells (CD8), Tregs, ILC2 cells, mast cells, M2 polarized macrophages, neutrophils, basophils, eosinophils, natural killer (NK) cells, and invariant natural killer T (iNKT) cells [17,19,23,24]. ST2 is also expressed in other cell types; however, its expression is inducible and depends on the cellular microenvironment. Although IL-33 is highly expressed at the mucosal tissue and in myofibroblasts, ST2 is mainly expressed on immune cells, allowing the IL-33/ST2 axis to act as a bridge between immune system orchestration and tissue injury, which is probably considered an essential component in intestinal immune responses [12]. IL-12 induces the ST2 expression on Th1 cells and on cytotoxic T cells, whereas IL-33 expression is critical for the activation of these cell populations [25,26]. IL-33 acts at both intracellular and extracellular levels. Intracellularly, IL-33 modulates the expression of various genes, acting as a nuclear factor [20]. Extracellularly, IL-33 operates as a cytokine, activating immune cells [20]. The human IL-33 contains an N-terminal nuclear localization signal (NLS) which controls the cytokine transfer to the nucleus, a central domain characterized as a “protease sensor” domain, and a C-terminal, IL-1-like region with cytokine activity [19,27,28]. Several proteases are accountable for the cleavage of the IL-33 within its central domain and produce the mature IL-33 form [29]. Conversely, cleavage of IL-33 by caspases into the IL-1-like domain during apoptosis leads to inactivation of the molecule [29,30]. This process reduces IL-33 biological activity, leading to the hypothesis that the presence of extracellular proteases can inactivate the full-length IL-33, averting potential detrimental effects induced by high circulating IL-33 levels [31]. However, in the microenvironment of inflammation, the N-terminal proteolytic cleavage by the proteases, neutrophil elastase and cathepsin G, is able to elevate its potency [32], underlying IL-33 in modulating the response to cellular damage.

IL-33 binds to its transmembrane receptor ST2, followed by a conformational alteration which results into the interaction of ST2 with the IL-1 receptor accessory protein (IL-1RAcP), a crucial molecule for IL-33 signaling [17,33]. The IL-33/ST2/IL1-RAcP complex is accountable for the Toll–interleukin receptor (TIR) dimerization [17]. This complex promotes intracellular signaling via the differentiation of MyD88, interleukin receptor-associated kinase (IRAK) 1 and 4, and tumor necrosis factor receptor-associated factor 6 (TRAF6) [17,27]. Through the aforementioned mechanisms, the mitogen-activated protein (MAP) kinases and NF-κB become activated, promoting the inflammatory cascade. In parallel, this complex induces the expression of extracellular signal-regulated kinase (ERK) and Jun kinase, which in turn promotes the downregulation of forkhead box p3 (Foxp3) and GATA3 transcription factors [20]. 

The significance of nuclear IL-33 sequestration and the great potency of IL-33/ST2 signaling in developing acute inflammation was presented by Carriere et al. The results of this study demonstrated that an alteration in the N-terminal part of IL-33 impeded the interaction of the cytokine with chromatin, resulting in the development of an inflammatory response, with splenomegaly, elevated lymph node influx, and colitis development [34]. Genetic ablation of ST2 led to cessation of the inflammatory response [34]. These findings indicate the role of IL-33/ST2 signaling as a bridge between tissue damage and orchestration of immune system, which may critically contribute to the maintenance of intestinal immunity.

### 2.2. IL-33/ST2 Signaling and Mucosal Immunity

Beyond the role of the IL-33/ST2 signaling pathway as a front-line herald of intestinal tissue damage, IL-33/ST2 also connects the innate and adaptive immunity with the host mucosal immunity, by inducing the type 2 response in T cells, ILCs and macrophages [35,36]. An interesting aspect of IL-33 is its role as an alarmin, acting at the barrier tissue, driving inflammation and fibrosis during acute mucosal damage [37]. The biologically active molecule of IL-33 is located in the nucleus bound to chromatin [34]. Cell lysis leads to the recruitment of neutrophils, eosinophils, and NK cells by IL-33, as well as proliferation of type 2 cells, thus commencing the fibrotic process and wound healing [38,39]. During this procedure, IL-33 also acts as a transcriptional factor and, through its binding to the p65 subunit of NF-κB, promotes the activation of endothelial cells [40].

## 3. IL-33 and IBD 

The maintenance of intestinal homeostasis is secured by the intestinal barrier proper function and the tolerogenic immune responses against commensal and beneficial microbes. As mentioned earlier, impaired barrier function and exaggerated immune responses to bacteria are critical contributing factors to IBD immunopathogenesis [3,7]. Breakdown of the intestinal barrier integrity and intestinal bacteria translocation lead to APC activation, including dendritic cells (DCs) and macrophages, which consequently result in proinflammatory cytokine production. Sensing of microbe-associated molecular patterns (MAMPs) by pattern recognition receptors (PRRs) leads to a vigorous release of proinflammatory cytokines and chemokines by innate immune cells residing in the gastrointestinal tract, resulting in Th1 and Th2 pathogenic responses [41]. Toll-like receptors (TLRs) and nucleotide-binding and oligomerization domain (NOD)-like receptors (NLRs) are able to recognize a wide array of PRRs that sense MAMPs [42].

**Figure 3:**
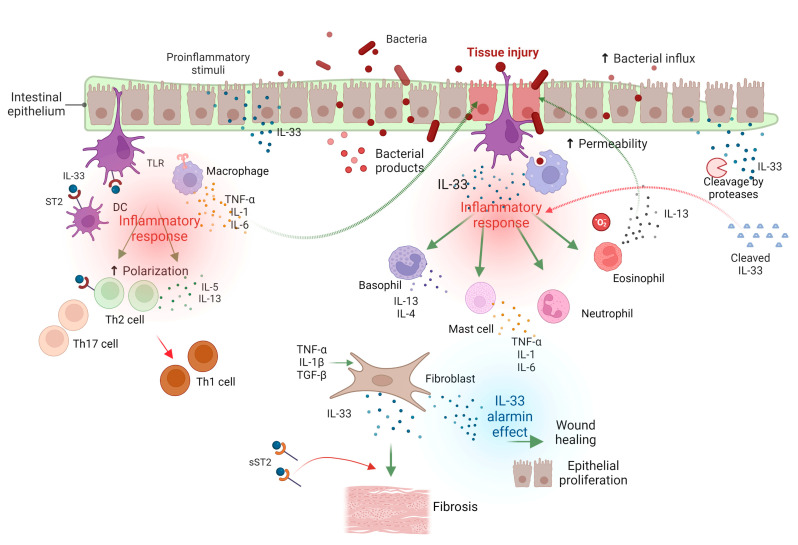
Role of IL-33/ST2 signaling pathway in inflammatory bowel disease pathogenesis. The intestinal epithelium of IBD patients is more exposed to pathogenic microorganisms, as the mucus layer is not able to obstruct pathogens’ access to the intestinal barrier, inducing epithelial impairment. In parallel, tissue damage can be provoked by infection or microbiota accumulation, which are also associated with IBD development. Epithelial damage results into the release of IL-33 from epithelial cells, inducing an inflammatory response. The activation of TLRs on the surface of macrophages leads to the secretion of proinflammatory cytokines, such as IL-6, TNF-α, and IL-1, which further induce epithelial injury. The presence of proinflammatory stimuli may also increase the intracellular IL-33 expression of intestinal epithelial cells. IL-33 activates the ST2-expressing cells to produce proinflammatory cytokines. IL-33 causes a polarization toward Th2 and Th17 immune responses. Eosinophil activation by IL-33 leads to IL-13 secretion, exerting detrimental effects on epithelial barrier integrity. Myofibroblasts in the intestinal tissue have also been reported as an IL-33 source in IBD. The mast cell-mediated inflammatory response may lead to fibroblasts proliferation toward fibrogenesis. However, the presence of sST2 could counteract the IL-33 cellular effect. IL-33, acting as alarmin in response to cellular stress induced by the mucosal breach, has also been shown to induce epithelial proliferation, repairing the epithelial barrier, and promoting wound healing. Biologically active IL-33 can be cleaved by proteases within the intestinal mucosa, alleviating the proinflammatory outcomes of IL-33. This figure was created with BioRender (https://biorender.com (accessed on 29 November 2022)). IL-33, interleukin-33; ST2, suppression of tumorigenicity 2; DC cells, dendritic cells; TLR, Toll-like receptor; TNF-α, tumor necrosis factor α; Th1, T helper 1; TGF-β, transforming growth factor-beta; sST2, soluble ST2.

IBD is induced by the exaggerated activation of NLRs and TLRs, followed by high expression of the proinflammatory cytokines IL-6, IL-12, IL-23, and tumor necrosis factor α (TNF-α) in the colonic mucosa by innate immune cells upon their activation with TLR ligands [43,44,45]. IL-33 and TLR-associated signaling commonly share the MyD88-dependent pathway, which activates downstream transcription factors [17,42]. Activation of ST2 signaling by IL-33 and MAMPs recognition through TLRs leads to NF-κB and MAP kinase activation [17]. The united action of IL-33 and TLRs promotes proinflammatory cytokine responses through the disruption of tolerogenic responses against intestinal bacteria [45] (Figure 3).

The role of IL-33 in IBD presents complexity; the divergent pathophysiology of the immune responses in IBD may account to some extent for this complexity. The immunological polarization of IBD has been classified as Th1- or Th2-related [46,47]. UC is related to a Th2 polarization [48], in contrast to CD, which has been associated with Th1 and Th17 polarization [49]; however, there are overlaps in immunological responses in these diseases [50]. 

## 4. IL-33 and Ulcerative Colitis

### 4.1. IL-33 Expression in UC-Affected Tissues 

The expression of the bioactive form of mucosal IL-33 is notably augmented in the intestinal epithelium and in infiltrating macrophages and B cells of the lamina propria of active UC patients, whereas, in serum samples, only the cleaved form of IL-33 has been detected [16]. A large number of studies have indicated that IL-33 transcripts and IL-33 protein are preferentially upregulated in the inflamed mucosa tissue of UC patients [14,51,52,53]. However, IL-33 expression has been also documented in ulceration-associated myofibroblasts of UC patients [52]. Likewise to IL-33, the expression of ST2 has been found elevated in both the colon wall and the serum of patients with IBD [14]. Even though the expression of ST2 in epithelium is reduced in IBD [16], an increase in infiltration of ST2-expressing APCs and T cells occurs in the lamina propria, and excessive infiltration of ST2-expressing immune cells is presented in the perivisceral adipose tissue of active IBD patients [31].

### 4.2. Detrimental Role of IL-33 

In the colonic mucosa, the IL-33/ST2 signaling pathway may present a dual and dichotomous role. Release of proinflammatory cytokines, such as IL-1β and TNF-α, and PAMP signals lead to increased expression of IL-33 in epithelial cells. Following epithelial injury, release of IL-33 may induce the immune responses by the ST2-expressing cells, alleviating the severity of inflammation [31,54], which has led to the speculation that IL-33 blockade in UC may exacerbate disease severity. In UC patients, the colonic mucosa tissue is characterized by increased activation of NKT and Th2 cells, followed by elevated production of IL-5 and IL-13 [43]. IL-33-mediated activation of ILC2 cells promotes the production of IL-5 and IL-13 [17]. Another subset of Th cells, the IL-9-producing Th9 cells, drives T-cell-mediated colitis via the IL-9 receptor signaling pathway in the intestinal epithelium [55]. IL-33 is closely implicated in the production of Th2 and Th9 cells [16,56]. Mice deficient in ST2 present resistance to dextran sodium sulfate (DSS)-induced colitis, whereas ST2 ablation promotes a wound-healing response following acute mechanical colonic injury, suggesting the contribution of the IL-33/ST2 axis to mucosal healing [56]. IL-33 secretion by the intestinal epithelium and myofibroblasts also results in increased Th9 cell stimulation, impairing intestinal barrier integrity, tissue repair, and immunological function [57]. In a DSS-induced acute colitis model, both ST2- and IL-33-knockout mice presented a delayed intestinal inflammatory response [58,59]. These results demonstrate a pathogenic role of IL-33 in the development of experimental UC via the impairment of intestinal barrier function and induction of Th2 responses. Mikulski et al. showed an early Th1 response followed by chronic Th2-mediated disease in a mouse model characterized by the development of T-cell-driven intestinal inflammation (SAMP mouse model) [60]. In the same model, a UC-like disease was associated with the expression of full-length IL-33 in the intestinal epithelium, while suppression of the IL-33/ST2 axis showed favorable outcomes [16]. These data indicate the dichotomous role of IL-33 in IBD, which may be defined by the pattern of T-cell response and the immunological differences in CD and UC.

### 4.3. Protective Role of IL-33 

On the other hand, IL-33-deficient mice presented high susceptibility to colitis and colorectal cancer, potentially suggesting a protective role as a mediator of intestinal immunity [61]. This finding provides a more complicated and contradictory role for IL-33 in IBD. Evidence is growing in line with this finding. IL-33 mRNA expression levels were reduced in biopsies derived from UC patients compared to the healthy controls, and a negative association was revealed between IL-33 expression and UC severity [62]. DSS-induced colitis is mostly T-cell-independent and is mediated by chemical injury, whereas the 2,4,6-trinitrobenzenesulfonic acid (TNBS) colitis model is dependent on stimulation of a Th1 immune response. IL-33, known for its close relation to inducing type 2 immunity, probably acts as a counteractor in TNBS-induced colitis. This is supported by a TNBS colitis model, in which administration of recombinant IL-33 resulted in reduced development of disease via the activation of M2-like macrophage polarization [63]. In parallel, IL-33-mediated improvement in a TNBS colitis model has been presented, dependent on Foxp3 expression, through the stimulation of Th2 and Treg immunity [64]. In the colonic tissue of active IBD patients, Tregs in the lamina propria have been found elevated compared to healthy individuals, and their function was normal [65]. IL-33 has been demonstrated to promote a TGF-β-mediated Foxp3^+^ Treg expansion in the intestinal tissue [66]. Specifically, Tregs expressed on colonic mucosa preferentially express ST2, and IL-33/ST2 signaling has been shown to induce the accumulation and maintenance of Tregs in the intestinal tissue and promote their protective activity [66]. However, the administration of recombinant IL-33 for enhancement of Treg-mediated protective function may be time-dependent, as IL-33 administration at initiation of DSS-induced colitis worsened disease severity. Recombinant IL-33 administration during the chronic or recovery phase improved DSS-induced colitis [67]. These findings could suggest that selective treatment of IL-33 in ST2-expressing Tregs may provide therapeutic advantages. A recent study presented IL-33-mediated tissue protection in a DSS colitis model, which was facilitated by ILC2 expression, in parallel with a Treg-supporting role [68]. This finding may be explained by the fact that ILC2s constitutively express the ST2 and, thus, could act directly following IL-33 treatment, compared to Tregs, in which only a subset expresses the receptor [68]. These results highlight a potential application of exogenous IL-33 in acute colitis concomitantly with a crucial role in inducing the stimulation of ILC2s to suppress intestinal inflammation.

The role of IL-33 may vary according to the disease stage. IL-33 has been found elevated in the serum of UC patients in the active phase compared to patients in remission [16]. Administration of recombinant IL-33 induced acute colitis but improved chronic colitis in a mice model via the mediation of amphiregulin/epidermal growth factor receptor (EGFR) signaling [69]. IL-33 enhanced neutrophil infiltration during both acute and chronic stages of the disease, which may be related to the aggravating effect on the acute stage by nitric oxide (NO) signaling. However, the results of this study showed that IL-33 decreased the translocation of pathobionts on the disrupted epithelium during the chronic stage of the disease [69]. These data highlight the essential role of differential IL-33 expression patterns in studying early and late events in immune responses, of IBD patients. Thus, beyond the role of IL-33 as an activator of acute inflammation, a protective role is also suggested for chronic inflammation, in long-term disease.

## 5. IL-33 and Crohn’s Disease

The production of proinflammatory cytokines such as IL-12, IL-23, and TNF-α by macrophages and DCs drives chronic inflammation in CD patients by promoting Th1 and Th17 immune responses. Release of IL-33 by myofibroblasts and intestinal epithelial cells induces the Th1 responses associated with CD [16,70,71]. IL-33 and IL-12, acting synergistically, induce the pathogenic Th1 responses [17]. Th1 cell differentiation is followed by an IL-12-dependent release of IL-33 by macrophages and DCs, upon exposure to commensal antigens and TLRs [43]. IL-12 secretion by DCs and macrophages located in the submucosa tissue of CD patients results in increased expression of ST2 via the stimulation of signal transducer and activator of transcription 4 (STAT4) [71]. Consequently, IL-33 is strongly involved in the development of pathogenic Th1 responses when antigens infiltrate the impaired intestinal epithelial tissue of CD patients [72]. This speculation is supported by the elevated expression of IL-33 which is associated with CD activity [16,56]. 

## 6. IL-33 and Intestinal Fibrosis

Intestinal fibrosis is a serious complication of IBD, mainly of CD patients [73]. During fibrosis development, certain intestinal parts become narrowed, markedly damaging the structure and function of the intestinal tract, leading to the need for endoscopic balloon dilation or surgery [74]. IL-33 induces the activation of critical cell populations mediating tissue fibrosis such as ILC2 cells and Th2 cells, thereby secreting profibrogenic factors [17]. Cytokines produced by Th2 cells, including IL-4, IL-5, and IL-13, promote pathological procedures such as an increase in mucous secretion, eosinophil influx, and tissue fibrosis [75]. In parallel, ILC2 cells also secrete type 2 cytokines via antigen-independent mechanisms, thus contributing to tissue fibrosis development [76]. These data were confirmed in in vivo models; specifically, IL-33 notably induced profibrogenic Th2 responses in a mixed Th1/Th2 model of IBD and the expansion of the ILC2 cells by IL-33 [16]. These procedures, followed by the production of IL-5 and IL-13, led to the development of intestinal fibrosis in the same experimental model [70]. In myofibroblasts of IBD patients, increased secretion of IL-33-induced IL-13 augments collagen accumulation through the suppression of matrix metalloproteinase synthesis [77], while IL-33 is able to directly induce the proliferation of human myofibroblasts [78]. These findings indicate the role of intestinal epithelium- and myofibroblast-produced IL-33 in inducing fibrogenic responses correlated with Th2 polarization.

## 7. Conclusions

ST2/IL-33 signaling in IBD has recently been highlighted as a critical subject of study. Cytokine networks involved in IBD pathogenesis present high complexity, and the use of related biological factors seems to exert favorable clinical outcomes in certain groups of IBD patients. The IL-33/ST2 axis interacts with important components of the intestinal tissue, including epithelial cells, gut microbiome, pathogenic bacteria, and immune cells. IL-33 is critically involved in IBD pathogenesis, acting as a modulator of mucosal immunity; however, its role is characterized as dual and dichotomous. IL-33 function is probably associated with the disease stage, as, although it enhances the early proinflammatory responses providing front-line protection against mucosal damage and pathogen infiltration, its baseline expression is critical for the proper function of intestinal barrier and integrity maintenance. Consequently, untimely or exaggerated secretion of the biologically active form of IL-33 may greatly contribute to IBD pathogenesis, indicating a great potential for therapeutic targeting.

## 8. Future Perspective

Accumulating evidence supports the pleiotropic nature of IL-33, which is further complicated by the fact that the IL-33/ST2 signaling axis presents extracellular and possibly intracellular functions, to block or induce IL-33 activity. Current research focuses on the elucidation of the underlying IL-33/ST2-associated mechanisms involved in IBD pathogenesis. A novel mechanism of IL-33 modulation by the microRNA 378a-3p (miR-378a-3p) in the intestinal colonic epithelium during inflammation was recently described. Inflammation-mediated suppression of miR-378a-3p resulted in elevated IL-33 protein levels in the intestinal epithelium, indicating a metabolic alteration that occurs in inflamed intestinal mucosal tissue [79].

Several points should be evaluated for the clarification of conflicting data on IL-33 activity in mucosal inflammation across various disease stages. In depth research into the post-transcriptional mechanisms modulating IL-33 activity, when interpreting the role of IL-33 in different disease settings, may highlight the relevant and/or differential role of this molecule in immunomodulation. The potential addition of novel IL-33-targeting biological agents in the therapeutic armamentarium against IBD necessitates the evaluation of “when” and “how” the IL-33/ST2 signaling should be targeted and whether it should be used combined with existing regimens.

## Data Availability

Not applicable.
